# Root Herbivore Effects on Aboveground Multitrophic Interactions: Patterns, Processes and Mechanisms

**DOI:** 10.1007/s10886-012-0104-z

**Published:** 2012-03-31

**Authors:** Roxina Soler, Wim H. Van der Putten, Jeffrey A. Harvey, Louise E. M. Vet, Marcel Dicke, T. Martijn Bezemer

**Affiliations:** 1 Laboratory of Entomology, Wageningen University, P.O. Box 8031, 6700 EH Wageningen, The Netherlands; 2 Department of Terrestrial Ecology, Netherlands Institute of Ecology (NIOO-KNAW), PO Box 50, 6700 AB Wageningen, The Netherlands; 3 Laboratory of Nematology, Wageningen University, PO Box 8123, 6700 ES Wageningen, The Netherlands

**Keywords:** Above-belowground interactions, Multitrophic interactions, Plant-insect interactions, Parasitoids, Plant defense

## Abstract

In terrestrial food webs, the study of multitrophic interactions traditionally has focused on organisms that share a common domain, mainly above ground. In the last two decades, it has become clear that to further understand multitrophic interactions, the barrier between the belowground and aboveground domains has to be crossed. Belowground organisms that are intimately associated with the roots of terrestrial plants can influence the levels of primary and secondary chemistry and biomass of aboveground plant parts. These changes, in turn, influence the growth, development, and survival of aboveground insect herbivores. The discovery that soil organisms, which are usually out of sight and out of mind, can affect plant-herbivore interactions aboveground raised the question if and how higher trophic level organisms, such as carnivores, could be influenced. At present, the study of above-belowground interactions is evolving from interactions between organisms directly associated with the plant roots and shoots (e.g., root feeders - plant - foliar herbivores) to interactions involving members of higher trophic levels (e.g., parasitoids), as well as non-herbivorous organisms (e.g., decomposers, symbiotic plant mutualists, and pollinators). This multitrophic approach linking above- and belowground food webs aims at addressing interactions between plants, herbivores, and carnivores in a more realistic community setting. The ultimate goal is to understand the ecology and evolution of species in communities and, ultimately how community interactions contribute to the functioning of terrestrial ecosystems. Here, we summarize studies on the effects of root feeders on aboveground insect herbivores and parasitoids and discuss if there are common trends. We discuss the mechanisms that have been reported to mediate these effects, from changes in concentrations of plant nutritional quality and secondary chemistry to defense signaling. Finally, we discuss how the traditional framework of fixed paired combinations of root- and shoot-related organisms feeding on a common plant can be transformed into a more dynamic and realistic framework that incorporates community variation in species, densities, space and time, in order to gain further insight in this exciting and rapidly developing field.

## Introduction

A central subject in terrestrial ecology is to understand the driving forces underlying the assemblage and functioning of plant-based communities. Within this field, the study of plant-insect interactions has played a pivotal role. Plant-insect interaction studies traditionally have focused on organisms that share a common domain, mainly aboveground. Aboveground herbivorous insects are the most speciose animal group on earth, and the intricate interactions with their host plants have fascinated ecologists for decades. In response to herbivory, plants often are defended by the production of or increase in the production of secondary plant compounds, phytotoxins, which impact the herbivore’s feeding activity and/or development. These plant defense responses often result in increased mortality, reduced growth rates and fitness of the attacker (Schoonhoven et al., 2005). Herbivorous insects, on the other hand, have evolved ways that detoxify such deleterious plant chemicals. Increased plant resistance in response to herbivory is called induced direct plant defense. Concentrations of plant defense compounds do not only occur locally in the leaf subjected to herbivory, but often increase in other leaves as well. Such a systemic response enables the protection of the still undamaged leaves from the herbivore. As a consequence, this response also can influence the performance of other organisms that are feeding from the same plant, but at other locations or later in time. In response to herbivory and egg deposition, plants also emit volatile secondary metabolites, which can be used by natural enemies of the herbivores, for example insect parasitoids, to locate their hosts (Dicke and Sabelis, 1988; Turlings et al., 1990; Vet and Dicke, 1992; De Moraes et al., 1998; Dicke, 1999; Fatouros et al., 2008). This response, known as induced indirect plant defense, is beneficial for parasitoids, because these detectable plant cues can indicate the presence of their ‘hard to detect’ hosts (Vet et al., 1991). The plants subsequently benefit from reduced levels of herbivory due to increased top-down control. The phytotoxins consumed by herbivores often accumulate in tissues such as fat body and hemolymph, and via this mechanism plants may also negatively affect the fitness of the developing parasitoid larvae that consume the host herbivore. This exemplifies how plant defenses can cascade up trophic chains in complex ways (Harvey et al., 2003). Because herbivore-induced direct and indirect plant defenses mediate interactions between species within and between trophic levels, across space and time, they are considered a central force in assembling plant-based communities (Kaplan and Denno, 2007).

In the field, plants also are exposed to belowground consumers. In many terrestrial ecosystems, root-feeding nematodes and insects are the dominant belowground attackers. In the early 1990’s, Masters et al. (1993) were among the first to report that root feeders can significantly alter interactions between plants and aboveground herbivores. This awareness of plant-mediated above-belowground interactions has brought a new level of complexity to the field of plant-insect ecology (Van der Putten et al., 2001; Bardgett and Wardle, 2003; Wardle et al., 2004). Interactive effects between plant consumers across domains have been explained by various induced plant responses, and a number of more recent studies indicate that these interactions often are mediated by herbivore induced plant defenses (reviewed in Bezemer and van Dam, 2005; Kaplan et al., 2008a; van Dam, 2009). In the early 2000’s, the question was raised whether and how changes within the plant induced by root herbivores could cascade up influencing parasitoids of foliar herbivores (Bezemer et al., 2005; Soler et al., 2005; White and Andow, 2006; Rasmann and Turlings, 2007). Other studies focussing on the effects of soil-dwelling plant mutualists have shown that, for example, arbuscular mycorrhizal fungi, plant growth-promoting rhizobacteria, and decomposers also can affect the growth and development of foliar herbivores and their level of parasitism (Masters et al., 2001; Van der Putten et al., 2001; Gange et al., 2003; Wurst and Jones, 2003; Guerrieri et al., 2004; Hempel et al., 2009; Pineda et al., 2010; 2012).

In the present review, we focus on the impact of root-feeding insects and nematodes on aboveground insect herbivores and their parasitoids; the effects of belowground symbionts are reviewed elsewhere in this issue (Jung et al. 2012, this issue). We first discuss the conceptual models that have been put forward to explain plant-mediated effects of root herbivores on aboveground insect herbivores; changes in plant nutritional quality and in secondary chemistry, from altered concentrations of foliar phytotoxins to defense signaling. The effects of root herbivory on higher trophic levels aboveground are comparatively less explored, and because general patterns cannot yet be drawn we discuss cases that exemplify the magnitude of these effects. We end by proposing that a way to advance this field is to study above-belowground interactions within a more dynamic and complex spatial-temporal approach that includes insect mobility and spatial and temporal aspects in experimental designs. A new approach that goes beyond the relatively static interactions between pairs of organisms forced to feed on the same plant at a single density and time.

## Impact of Root-Feeding Insects on Foliar Herbivores

**Fig. 1:**
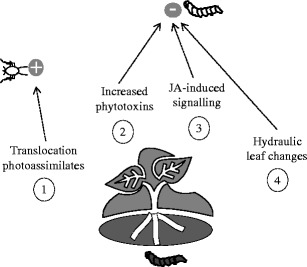
*Plant-mediated effects of root-feeding insects on aboveground leaf chewers and phloem feeders.* The aphid (left) represents aboveground phloem feeders, and the white caterpillar (right) represents leaf chewers. The grey caterpillar represents root-feeding insects. Effects of root herbivory can be positive (+) or negative (−) for overall aboveground insect performance, relative to insects on undamaged plants. Mechanisms that have been put forward to explain these plant-mediated effects are induced changes in shoot nutritional quality (1), shoot secondary chemistry (2 and 3), and hydraulic leaf changes (4). Numbers indicate each of the proposed hypotheses discussed in the text

Quantitative reviews show that in the vast majority of cases, insect herbivores that feed from the same plant affect each other negatively (Denno et al., 1995). These plant-mediated competitive interactions often are caused by increases in secondary plant compounds induced by the initial attacking species that negatively affect the subsequent species (Kaplan and Denno, 2007). In Fig. 1, we summarize the main patterns and mechanisms that have been proposed to explain the, positive and negative, effects that root-feeding insects can have on the survival, fecundity, growth and/or development of aboveground insect herbivores. One of the earliest aboveground-belowground studies reported a positive effect of root-feeding insects on the performance of aboveground aphids, and attributed this facilitation to an improvement in shoot nutritional quality measured as increases in total soluble nitrogen (Gange and Brown, 1989). Later studies further confirmed that aphids perform better when feeding on plants previously colonized by root-feeding insects compared to uninfested plants (Moran and Whitham, 1990; Masters and Brown, 1992). Based on these results, Masters et al. (1993) proposed the first mechanistic hypothesis linking spatially separated herbivores, the ‘Stress Response Hypothesis’ (Fig. 1, ①). According to this hypothesis, the capacity of roots to acquire water and nutrients from the soil is constrained due to removal of root tissue. This creates an effect within the plant similar to water stress, leading to the accumulation of soluble nitrogen and carbon in the foliage, facilitating the growth and development of the herbivores. This hypothesis has been derived from the ‘Plant Stress Hypothesis’, which predicts that plants subjected to non-extreme abiotic stress, for example water limitation, shading or pollution, become more susceptible to herbivores due to a temporal increase in the amount of soluble nitrogen that is mobilized from the site of attack to sites of storage and new growth (White, 1984). More recent studies that also observed positive effects of root herbivory on aphid performance, did not find significant differences in concentrations of soluble nitrogen in plants with or without root-feeding insects (Johnson et al., 2009). It is noteworthy that during the last one or two decades various meta-analyses have shown that water stress in plants frequently does not lead to increased performance of aphids (Koricheva et al., 1998; Huberty and Denno, 2004), which further challenges this hypothesis.

**Fig. 2:**
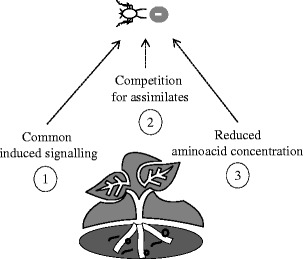
*Plant-mediated effects of root-feeding nematodes on aboveground aphids.* The aphid represents aboveground phloem feeders, and the black circles and curved lines represent ecto- and migratory endoparasitic nematodes and root-knot or cyst-forming nematodes, respectively. Effects of herbivory by nematodes on aphid fitness are mostly negative (−) relative to that on undamaged plants. Mechanisms that have been put forward to explain these negative effects are induction of common defense signaling (1), competition for assimilates in the phloem (2), and reduced amino acid concentration in the phloem (3). Numbers indicate each of the proposed hypotheses discussed below

Other studies that have examined the effects of root herbivores on aboveground leaf chewers have reported negative effects, showing that besides facilitation, plant-mediated competition also is common in aboveground-belowground interactions (Tindall and Stout, 2001; Bezemer et al., 2003; van Dam et al., 2003, 2005; Soler et al., 2005; Staley et al., 2007). The frequently observed negative impact of root herbivory on leaf chewer fitness has been explained by the ‘Defense Induction Hypothesis’ (Bezemer et al., 2003). This hypothesis states that above- and belowground insect herbivores influence each other via induced changes in secondary plant compounds (Fig. 1, ②). Insects that feed from the phloem are less exposed to secondary plant compounds, since phytotoxins generally are stored in cells (Larsson, 1989). This can explain why aboveground aphids usually are not negatively affected by root herbivory. In this view, root-chewing insects induce an increase in foliar secondary plant compounds, which negatively affects the performance of leaf chewers without affecting phloem feeders (reviewed in Bezemer and van Dam, 2005; Johnson et al., 2008; Kaplan et al., 2008a; van Dam and Heil, 2011).

**Fig. 3:**
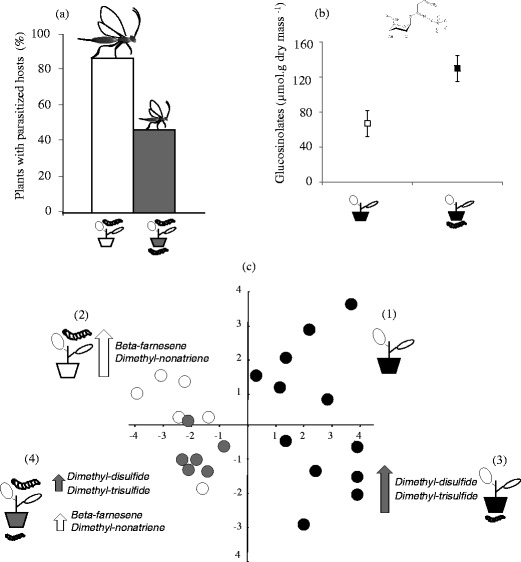
*Root-feeding insects and aboveground parasitoids. A case study.* **a** Percentage of *Brassica nigra* plants with foliar-feeding *Pieris brassicae* hosts selected for oviposition by females of the parasitoid *Cotesia glomerata*. The size of the parasitoid reflects its relative performance on plants without (*white bars*) and with (*grey bars*) *Delia radicum* root-feeding larvae. **b** Glucosinolate (*sinigrin*) level in young leaves of *B. nigra* plants (*white dotted squares*) and plants infested by *D. radicum* (*grey squares*). **c** Canonical discriminant plot showing sample scores based on volatile blends of *B. nigra* plants (1) without herbivores (2) with *Pieris brassicae* larvae, (3) with *Delia radicum* larvae and (4) with both herbivores. Each circle represents a sampled plant. Beta-farnesene and dimethyl-nonatriene are known attractant compounds (*white arrows*) for insect parasitoids, while sulfides are known repellent volatiles (*grey arrows*) for insects; the size of the arrows represents the relative amount of the compounds in the blends of the plants with root- and foliar-feeding insects. Summary from R. Soler PhD Thesis, Netherlands Institute of Ecology, 2007 (reprints of the thesis can be requested by e-mail)

There has been a significant development in the understanding of the molecular mechanisms underlying local and systemic induced plant defenses triggered by pathogens and insects aboveground (Kessler and Baldwin, 2002; Zheng and Dicke, 2008; Pieterse et al., 2009). This has enabled the exploration of induced plant defenses beyond measuring changes in nutrients and phytotoxins, thus providing a basis to mechanistically understand plant-mediated interactions. Generally, leaf-chewing insects such as caterpillars cause a response in the plant that triggers the jasmonic acid (JA) signaling pathway, while phloem-feeding insects such as aphids induce the salicylic acid (SA) signaling pathway. Although the majority of studies have focused on signaling responses in the foliage in response to shoot attack, these responses also occur in the roots (reviewed in Erb et al., 2009a). It has been shown that jasmonates can be transported from shoots to roots (Baldwin et al., 1994), showing how long distance defense signaling can occur across roots and shoots. The transport of jasmonates from roots to shoots can explain why root-feeding insects may negatively impact the performance of foliar insect herbivores, because JA in the roots is transported to/activated in the shoots (Fig. 1, ③).

Jasmonic acid and salicylic acid often act antagonistically, and increases in the levels of one of the phytohormones can interfere with the activity of other phytohormones (Pieterse and van Loon, 1999; Engelberth et al., 2001; Kessler and Baldwin, 2002; Koornneef et al., 2008; but see e.g., Schenk et al., 2000; Van Wees et al., 2000 that report synergistic interactions). If this so-called cross-talk between pathways (Pieterse et al., 2009) also occurs across plant organs, root herbivory can cause a reduction in SA- related defenses in the foliage by inducing JA-related defenses as proposed by Van der Putten et al. (2001). This can provide an alternative explanation for the frequently observed increased performance of phloem feeders on plants previously attacked by root-feeding insects. However, in *Zea mays* (maize) plants, neither JA nor SA were found to be induced in the shoots by the rootworm *Diabrotica virgifera* (Erb et al., 2009b). Interestingly, leaves of root-infested maize plants had reduced leaf water contents and increased levels of abscisic acid (ABA) (Erb et al., 2011a).

Reduced resistance to leaf chewers has been reported on ABA-deficient plants (Thaler and Bostock, 2004; Bodenhausen and Reymond, 2007), leading the authors to hypothesize that, in *Z. mays*, increased resistance to leaf chewers in plants with root herbivory is due to induced ABA signaling and/or hydraulic changes in the leaves (Erb et al., 2011a). Abscisic acid is involved in a number of physiological adaptations of plants to drought stress, and it can act as a chemical signal that controls the opening and closing of stomata. It might be difficult then to disentangle the effects of changes in ABA and leaf water content on foliar herbivores. Interestingly, the negative effects on the leaf chewer were still observed after ABA signaling was inhibited. More studies that explore defense signaling that cross the border between the below- and aboveground domains are needed to understand the mechanistic basis that mediate these interactions (Erb et al., 2009a).

Knowledge about the molecular mechanisms underlying plant defenses is derived from a limited number of model plants species from genetic and molecular biology (Felton and Korth, 2000; Stout et al., 2006; Wang et al., 2008; but see Wu and Baldwin, 2010; Broekgaarden et al., 2010), and often herbivory is simulated by using exogenous applications of JA and SA (e.g., Spoel et al., 2003; Koornneef et al., 2008; Leon-Reyes et al., 2010; but see e.g., Kessler et al., 2004). Consequently, extrapolations into ecologically representative scenarios have to be taken with caution. Studies with natural communities are needed to determine the full ecological and evolutionary consequences of above-belowground multitrophic interactions.

## Impact of Root-Feeding Nematodes on Foliar Herbivores

Root-feeding nematodes are dominant belowground herbivores and important pests worldwide. They are the main group of root herbivores in temperate grasslands and their feeding activities can affect aboveground plant size and nutritional quality (Stanton, 1988). The impact of root-feeding nematodes on aboveground insects has been less well-studied than the effects of root-feeding insects. However, an increasing number of studies are showing that root-feeding nematodes also can influence aboveground insects via their effects on the shared host plant (e.g., Bezemer et al., 2005; Kaplan et al., 2011). In Fig. 2, we summarize the most commonly observed effects, and discuss potential mechanisms to explain these linkages.

In contrast to root feeding by insects, which often facilitate the growth and development of aphids, studies on feeding by nematodes consistently report negative effects on aphid performance (Bezemer et al., 2005; Wurst and Van der Putten, 2007; Kaplan et al., 2009, 2011; Hol et al., 2010; Vandegehuchte et al., 2010; Kabouw et al., 2011). Nematode-caterpillar interactions are less well-studied, and positive (Alston et al., 1991; Kaplan et al., 2008b), neutral (Wurst and Van der Putten, 2007), and negative effects (van Dam et al., 2005) have been reported. We will, therefore, focus on the mechanisms that have been proposed to link the consistent negative impact of nematodes on aphid fitness. The first proposed explanation was that nematodes and phloem feeders trigger a common defense signaling pathway (Kaplan et al., 2009). This hypothesis is based on studies that showed that in Solanaceae, the defense gene Mi-1 mediates resistance to both root-knot nematodes and aphids (Li et al., 2006; Bhattarai et al., 2007). Thus, aboveground phloem feeders and root-feeding nematodes might be inducing similar defense pathways in plants (Fig. 2, ①). Subsequent studies have shown that although Mi-1 mediates resistance to both nematodes and phloem feeders/sap suckers, it is involved in the activation of distinct signaling pathways. Therefore, the Mi-1 defense gene may contribute differently to the resistance to aphids and nematodes (Mantelin et al., 2011). There is no empirical evidence yet that links the reduced performance of phloem feeders on plants exposed to nematodes with changes in levels of phytohormones or defense marker genes.

More recently, Kaplan et al. (2011) empirically tested the ‘Sink Competition Hypothesis’, which proposes that aboveground phloem feeders and root-feeding nematodes compete for assimilates in the phloem. Root-knot nematodes and aphids feed from vascular tissues and attract photoassimilates to their feeding site. Therefore, the pressure-driven transport in the phloem sieve elements can be re-directed towards root-feeding nematodes or aphids, and thus both can act as a nutrient sink for the plant (Guerrieri and Digilio, 2008). Thus, when nematodes colonize the roots of the plant earlier than aphids, the sink created by nematodes in the roots may compete with the subsequent sink that aphids will initiate in the shoots (Fig. 2, ②). Empirical evidence for this potential mechanism is lacking (but see Inbar et al., 1995; Larson and Whitham, 1997 for evidence supporting the hypothesis in aboveground plant-herbivore interactions). Especially cyst- or gall-forming species are able to feed from the phloem, which makes them potential competitors of aphids. It is noteworthy that aphids also perform suboptimally on plants infested by migratory endoparasitic species that do not create nutrient sinks within the plant (e.g., Wurst and Van der Putten, 2007). The concentration of amino acids in the phloem of plants infested by root-feeding nematodes also has been reported to be lower than on plants without nematodes, and this change correlated with the reduced aphid fitness that was observed (Bezemer et al., 2005). More studies are needed to confirm how widespread this mechanism is.

## Root Feeders and Aboveground Parasitoids: Potential Interactions

### Interactions via Changes in Herbivore Induced Plant Volatiles

In the early 2000’s, the question was raised whether soil-dwelling organisms also could affect parasitoids of aboveground herbivores. The first studies focused on parasitoid host-plant preferences, and all reported that the level of attraction of female parasitoids was increased when plants were exposed to soil-dwelling organisms, independently of the soil functional group triggering the effect. Therefore, it was proposed initially that soil organisms, independent of whether they were root antagonists or plant beneficials, would all benefit host-parasitoid interactions (e.g., Masters et al., 2001; Gange et al., 2003; Wurst and Jones, 2003; Guerrieri et al., 2004). However, a potential mechanism responsible for the increase in host plant preference was not provided in these studies. Considering that in aboveground systems, parasitoid host-searching is guided primarily by volatile cues that are produced by the host-infested plant (Dicke, et al., 1990; Turlings, et al., 1990; Vet and Dicke, 1992), herbivore-induced plant volatiles were a primary candidate to test. Subsequent studies have shown that the composition of the volatile blend induced by foliar herbivores can be affected by root-feeding insects. The result is that the plant becomes less attractive to female parasitoids foraging for hosts (Rasmann and Turlings, 2007; Soler et al., 2007a). In these studies, root-feeding by insects clearly interfered with host-parasitoid interactions. Other studies also have shown that volatiles emitted by plants exposed to both foliar- and root-feeding insects can be quantitatively and qualitatively different from blends emitted by plants exposed to each herbivore in isolation (Olson et al., 2008; Pierre et al., 2011). It is well-established that specialist parasitoids can distinguish between plants attacked by their hosts and plants attacked by non-hosts by exploiting differences in induced plant volatiles (de Moraes et al., 1998). It is less clear, however, what can happen when the same plant is exposed to multiple host and non-host herbivores of the parasitoid (but see Shiojiri et al., 2001, 2002; Vos et al., 2001; Rodriguez-Soana et al., 2002; 2005; Zhang et al., 2009; Dicke et al., 2009; Erb et al., 2010), especially when these herbivores feed from roots and shoots.

### Interactions via Changes in Host Quality and Consequences for Parasitoid Behaviour

Parasitoid larvae are highly susceptible to changes in the quality of the internal biochemical environment provided by their hosts, and thus are tightly linked to host development (Harvey, 2005). As root herbivores can influence the growth and development of aboveground insect herbivores via induced changes in foliar secondary chemistry, these effects also could affect the developing parasitoid larvae. A number of studies have shown that root herbivore effects can even be stronger for the developing parasitoid larvae than for the herbivore itself (Bezemer et al., 2005; Soler et al., 2005, but see Kabouw et al., 2011 where no effects were observed). These effects can cascade up to at least the fourth trophic level influencing hyperparasitoid fitness (Soler et al., 2005).

Unlike predators, which frequently consume multiple prey individuals, the resources available for parasitoid development are restricted to a single host. Consequently, parasitoids are under strong selection pressure to optimize usage and disposal of these limited resources (reviewed in Harvey, 2005). Optimal foraging theory predicts that carnivores choose to attack host/prey species that are most rewarding for them in terms of their fitness (Krebs and Davies, 1984). Similarly, within a host species, parasitoid females are expected to select the most profitable individuals that maximize their fitness (Godfray, 1994). Since the adequacy of foraging choices of parasitoids is linked directly with their reproductive success, females can be expected to select in favor or against hosts feeding on plants already infested by root herbivores, depending on how root herbivory affects the performance of the parasitoid. Most studies that link above-belowground multitrophic interactions address either effects on parasitoid attraction or changes in plant volatiles but not both. Therefore, it remains unclear how common it is that root herbivory affects aboveground host-parasitoid interactions by changes in plant volatile emission. In Table 1, we summarize studies that have addressed these aspects.

**Table 1:** Effects of root feeding insects (a) and nematodes (b) on parasitoid performance, behavior, and/or changes in plant volatiles. Rch: root-chewer, rk: root-knot, sp: seed predator, lch: leaf chewer, and pf: phloem feeder

Root herbivores	Plant species	Foliar herbivores	Parasitoids	Performance-related effects	Behavioural-related effects	HIPV changes	Reference
(a) Insects							
General insects	*Cirisum palustre*	*Terellia ruficauda* (sp)	*Pteromalus elevatus Torymus chloromerus*		Increased population abundance		Masters et al., 2001
*Delia radicum* (rch)	*Brassica nigra*	*Pieris brassicae* (lch)	*Cotesia glomerata*	Reduced size			Soler et al., 2005
				Longer development			
*Diabrotica virgifera* (rch)	*Zea mays*	*Ostrinia nubilalis* (lch)	*Macrocentrus grandii*		Reduced population abundance		White and Andow, 2006
*Delia radicum* (rch)	*Brassica nigra*	*Pieris brassicae* (lch)	*Cotesia glomerata*		Attraction attenuation	More repellent + less attractants	Soler et al., 2007a
*Delia radicum* (rch)	*Brassica nigra*	*Pieris brassicae* (lch)	*Cotesia glomerata*		Reduced searching efficiency		Soler et al., 2007b
*Diabrotica virgifera* (rch)	*Zea mays*	*Spodoptera littoralis* (lch)	*Cotesia marginiventris*		Attraction attenuation		Rasmann and Turlings, 2007
					Associative learning		
*Agriotes* spp. (rch)	*Plantago lanceolata*	None	None			No observed effect	Wurst et al., 2008
*Delia radicum* (rch)	*Brassica rapa*	*Pieris brassicae* (lch)	None			Qualitative + quantitative differences	Pierre et al., 2011
(b) Nematodes							
General Nematodes	*Agrostis capillaris*	*Rhopalosiphum padi* (pf)	*Aphidius colemani*	Reduced mortality			Bezemer et al., 2005
	*Anthoxanthum odoratum*						
*Meloidogyne incognita* (rk)	*Gossypium* spp.	*Heliocoverpa zea* (lch)	*Microplitis croceipes*		No observed effect	Increased levels	Olson et al., 2008
General nematdes	*Brassica oleracea*	*Brevicoryne brassicae* (pf)	*Diaeretiella rapae*		No observed effect		Kabouw et al., 2011

In Fig. 3, we summarize case studies that provide support for the hypothesis that the degree of preference of female parasitoids for hosts feeding on plants already infested by root herbivores will depend on how root herbivory affects the performance of their offspring (Soler et al., 2005, 2007a). *Cotesia glomerata* females parasitized significantly more *Pieris brassicae* hosts on *Brassica nigra* plants without than with the root herbivore *Delia radicum* (Fig. 3a). Parasitoids also developed significantly better on hosts that were feeding on plants without root herbivory (Fig. 3a). In the presence of root herbivory, the amount of sinigrin, which represented 99% of the total glucosinolate contents in the shoots of *B. nigra*, was significantly higher (Fig. 3b). The suboptimal parasitoid performance in root-infested plants was attributed to the increased sinigrin concentration in shoots of plants with root herbivores. This behavior shows a clear preference-performance linkage for the parasitoid that will enhance the performance of its offspring. The volatile blends emitted by undamaged plants, by plants damaged by *Pieris brassicae* (the leaf-chewing host of the parasitoid), by plants exposed to *Delia radicum* (the root herbivore), and by plants exposed to both types of herbivory differed significantly (Fig. 3c). Plants exposed to the leaf chewer were characterized by high levels of beta-farnesene and dimethylnonatriene, which are volatile compounds reported to act as attractants for herbivorous and carnivorous insects (Dicke et al., 1990; Fukushima et al., 2002; Ansebo et al., 2005). In contrast, plants exposed to root herbivory were characterized by high amounts of sulphides, such as dimethyl disulfide and dimethyl trisulfide, which act as repellents/toxins to insects (Dugravot et al., 2004). The reduced preference of female parasitoids for hosts feeding on plants colonized by root-feeding insects may be attributed to the relatively high levels of repellents and low levels of attractants that root and shoot co-infested plants emit compared to conspecific plants with only hosts. Taken together, these results suggest that root-damaged plants convey chemical information that aboveground parasitoids can use to optimize oviposition decisions (but see Olson et al., 2008). This expectation is confirmed by these studies, but support for this hypothesis remains scarce.

Innate responses of foraging parasitoids to plant odors can change with experience, leading to local or temporary specialization and enhancement of foraging success (Turlings et al., 1990; Vet et al., 1995). Parasitoids have the ability to learn to distinguish between volatile blends emitted by plants infested by their hosts *versus* plants infested with their hosts and root-feeding insects (Rasmann and Turlings, 2007). Therefore, they could regain attraction for hosts feeding on root-infested plants with experience (Rasmann and Turlings, 2007). Yet, the effects of parasitoid learning in this process need to be explored. The role of parasitoid learning in dealing with natural variation in plant and host quality and plant volatiles induced by root herbivory remains largely unstudied.

## Incorporating Community Variation in Species, Densities, Space, and Time

Thus far, the majority of above-belowground interaction studies that involve plants, insects, mutualistic symbionts, and natural enemies have encompassed relatively little variation in number of players and in environmental conditions. Here, we review studies that are extending this scope by bringing in effects of time, space, behavior, and habitat conditions. We identify this as the direction of future studies in the area of above-belowground multitrophic interactions.

### Time of Arrival of Root and Shoot Herbivores

The sequence of arrival of above- and belowground herbivores on a plant can greatly affect the outcome of the interaction (Maron, 1998; Blossey and Hunt-Joshi, 2003). The leaf chewer *Spodoptera fugiperda*, for example, had a significant negative effect on the colonization of the root chewer *Diabrotica virgifera* when first colonizing the plant, but the aboveground herbivore did not influence the performance of the root feeder when arriving later than the root herbivore (Erb et al., 2011b). The sequence of arrival also has been shown to be an important determinant of plant responses at the gene level. Transcriptional changes, for example, have been shown to differ significantly for sequential and simultaneous attack of aboveground leaf chewers and phloem feeders (Voelckel and Baldwin, 2004). Similarly, the expression of SA- and JA-related genes has been found to differ in response to individual and simultaneous shoot attack by insect herbivores from contrasting feeding-guilds (Zhang et al., 2009; Soler et al., 2012). Aboveground insect herbivores that feed on a plant already infested by root feeders are expected to be inevitably confronted with higher levels of phytotoxins, and thus potential fitness costs (Bezemer and van Dam, 2005). This idea is based on studies with *Gossypium herbaceum*, cotton plants, that showed that in response to root herbivory levels of secondary compounds increased along the entire shoot (Bezemer et al., 2004). However, it is not clear how widespread this response can be. For example, a subsequent study in which *B. nigra* plants were exposed to root herbivory showed that levels of secondary compounds were increased only in young leaves in response to root feeding, but that they did not change in mature and old leaves (Soler et al., 2005). More studies that record changes in secondary chemistry in response to root herbivory that compare both young and old leaves are needed to determine how common this phenomenon is.

### Spatial Distribution of Root Feeders

Besides the mere presence or absence of root feeders on the plant, the spatial distribution of root-infested plants in a habitat can be of crucial importance. Evidence for this assumption is provided by a field study where the specialist aphid *Brevicoryne brassicae* preferred to feed and reproduce on *B. nigra* plants without root herbivores over plants infested by the root herbivore *D. radicum*. This preference was observed only when plants with root herbivores were grouped in clusters. When the plants with and without root herbivores were placed in a mixed design, aphids no longer differentiated (Soler et al., 2009). This shows that the spatial arrangement of root herbivores in the field also can be an important factor determining the amount of aboveground herbivory. However, as discussed in the previous section, it remains unknown whether root feeders uniformly influence the secondary chemistry of the entire shoot or if these changes are restricted to certain parts of the shoot. In response to aboveground insects, for example, phytotoxins often increase in certain tissues, e.g., young leaves, rather than uniformly along the shoot, thus allowing secondary attackers to scape potential fitness costs by avoiding feeding on theses leaves (Stout et al., 1996). When root induced plant responses are expressed only in certain parts of the shoot, only the aboveground herbivores that feed on these parts are expected to be influenced by root feeders (Kaplan et al., 2008c).

### Herbivore and Parasitoid Preferences

Most above-belowground studies are based on non-choice experiments where the survival, growth, and development of caterpillars or aphids on plants with or without root herbivores are compared. Foliar herbivores, however, can precisely select plants for oviposition and feeding. Where free choices can be made, aboveground insect herbivores can avoid or prefer plants that are already colonized by root feeders. Optimal oviposition theory predicts that females of herbivorous arthropods with offspring with limited mobility, such as butterflies, will evolve to select those host plants for oviposition on which their offspring perform best thus maximizing their fitness (Jaenike, 1990). Considering that plants attacked by root-feeding insects often represent a suboptimal food source for leaf chewers, butterflies should avoid plants with root herbivores and select uninfested conspecifics if these represent fitness costs (Soler et al., 2010). When such avoidance occurs, this also will be beneficial for the plant by reducing the probability of root-damaged plants being simultaneously attacked belowground and aboveground. The same approach might apply belowground, and there are studies, for example on root-feeding nematodes, where the presence of potential enemies may direct attackers away from potential feeding sites (Piskiewicz et al., 2009).

Adding effects on the reduced preferences that natural enemies of herbivores can show for hosts feeding on plants also attacked by root herbivores (Rasmann and Turlings, 2007; Soler et al., 2007a) will show the complex dimensions of the ecological ‘dilemma’ for leaf-chewing insects with respect to root-infested host plants. The evolutionary choice would be between growing more slowly and/or attaining a smaller size but benefitting from a smaller probability of being found by natural enemies on root-infested plants, or optimizing performance at the cost of running a higher risk of parasitism or predation on root-uninfested healthy plants. From the plant’s point of view, the benefits of acting as a communication channel between root- and foliar-feeding herbivores that attenuates simultaneous infestations is then counterbalanced by interferences with the indirect defense system of the plant that reduces the attraction of natural enemies of the herbivore. If and how above- and belowground herbivores may integrate all this information in their “decision-making” remains to be elucidated.

### Parasitoids and Effects Through Changes in the Habitat

Interactions between root feeders and parasitoids are not restricted to interactions on a single plant. For example, root herbivores can influence host-parasitoid interactions aboveground via their effects on changes in the structure of the plants. In *Z. mays*, the percentage of parasitism of the European corn borer, *Ostrinia nubilalis*, by its specialist parasitoid *Macrocentrus grandii* was significantly reduced in the presence of the corn rootworm *Diabrotica virgifera* in the habitat (White and Andow, 2006). Plant height and density were reduced in habitats where the rootworm was present, resulting in more open habitats that are less preferred by female parasitoids of this species. Interestingly, this positive indirect interaction, known as associational resistance, in which one species gains protection from its consumer by association with a competitor, has been widely documented in plants (Andow, 1991), but not among insects. Root herbivores also can influence host-parasitoid interactions aboveground via changes in the quality of the surrounding environment triggered by belowground insects. Females of the parasitoid *Cotesia glomerata* found their hosts on focal plants much faster in situations when neighboring plants were exposed to root herbivory, than when neighboring plants were kept undamaged (Soler et al., 2007b). In that study, the microhabitat was composed of root-damaged and root-undamaged plants of the same species that all had similar size and height, which minimizes the influence of physical plant characteristics on the foraging wasps (McCann et al., 1998; Gols et al., 2005).

### Plant-Mediated Aboveground-Belowground Interactions in the Field

A number of studies have shown that the abundance or preference of aboveground organisms, such as herbivores, pollinators, predators, or parasitoids, on plants growing in natural or agricultural systems can be affected by whether the plant is also exposed to root herbivory (e.g., Masters, 1995; Poveda et al., 2003; Hunt-Joshi and Blossey, 2005; Staley et al., 2007; Wurst et al., 2008; Kaplan et al., 2009; Soler et al., 2009). Most of these studies have used potted plants with or without root herbivory that are placed in the field (e.g., Poveda et al., 2003; Wurst et al., 2008; Soler et al., 2009). However, several studies have manipulated aboveground and belowground herbivory in the field that show that root herbivory by insects or nematodes can affect aboveground multitrophic interactions under natural conditions (e.g., Blossey and Hunt-Joshi, 2003; White and Andow, 2006; Kaplan et al., 2009), while others have not detected a significant effect (Hladun and Adler, 2009; Hong et al., 2011; Heeren et al., 2012). Interestingly, two recent independent studies report that there are no significant interactions between soybean cyst nematodes and aphids in soybean fields (Hong et al., 2011; Heeren et al., 2012). In contrast, greenhouse studies with soybean plants have reported that the performance of soybean aphids is significantly influenced by cyst nematodes (e.g., Hong et al., 2010). These results indicate that care needs to be taken when extrapolating results from greenhouse and common garden experiments to real field situations, and emphasize the urgent need for more realistic above-belowground studies.

### Belowground Influences of Aboveground Induced Defenses in the Field

Another issue that remains largely unresolved is how important the effects of root herbivory on aboveground induced plant defense responses are for plants that are growing in the field and are interacting with multiple antagonists, mutualists, decomposers, and other plants simultaneously. Most field studies that examine root herbivore effects on aboveground plant-insect interactions do not report effects on secondary plant compounds or emission of volatiles. However, a recent study by Megias and Muller (2010) shows that exposure to root herbivory in field-grown brassicaceous plants (*Moricandia moricandioides*) led to significant changes in aboveground glucosinolate profiles, and that these differences correlate with changes in the composition of the aboveground food web on these plants. This study shows clearly that root induced changes in aboveground plant secondary compounds can be of significant importance in the field. Similarly, Hladun and Adler (2009) showed that *Cucurbita moschata* plants, butternut squash, grown in the field had increased floral nectar concentrations when exposed to root herbivory. This can subsequently affect pollinators, but also parasitoids and predators in the field. As there is now a considerable number of studies that have shown that levels of parasitism and predator abundance in the field can be affected by root herbivory (e.g., Masters et al., 2001; White and Andow, 2006; Soler et al., 2009), it is quite possible that root herbivory indeed affects aboveground indirect induced defense responses in the field. Further field-based studies are needed in order to determine how these interactions can influence, or are influenced by, species diversity and community structure. How important indirect plant defense responses can be in the field (Obermaier et al., 2008), and how this is affected by root herbivory remains to be explored.

## Concluding Remarks

It is evident that root feeders can be important players in aboveground plant-based communities, via their effects on direct and indirect defenses of plant shoots that can cascade up to at least the fourth trophic level. Knowing this, the new challenge is to study above-belowground interactions under more realistic conditions. This will bring us closer to the detection of mechanisms with evolutionary potential and patterns that can be used in practice, for example when attempting to enhance sustainable pest control. It is puzzling why root-feeding insects and nematodes are still playing a minor role in the studies of contemporary community, behavioral, chemical, and molecular ecology. Currently, the notion of ‘out of sight, out of mind’ is no longer a valid argument for leaving out root feeders!
